# Supplementary material for: Multi-angle laser device improves novice learning of C-arm fluoroscopy for lumbar spine surgery
Source: PLoS One. 2024 Mar 7;19(3):e0299273. doi: 10.1371/journal.pone.0299273 (PMC10919629; doi:10.1371/journal.pone.0299273)
Supplement: Attachment — Submitted filename: Manuscript # PONE-D-23-30166 revision.docx [file pone.0299273.s002.docx]

The manuscript titled “Multi-angle laser device improves novice learning of C-arm fluoroscopy for lumbar spine surgery” by Yuan-Dong ZHUANG^1#^, Rui-Jin LI^2#^ et al. investigates the use of multi-angle laser device (MLD) for C-arm fluoroscopy to assist novice learners during lumbar spine surgery. The findings of the authors suggest that the MLD significantly improves novice learning of C-arm fluoroscopy during lumbar spine surgery. The study's findings are intriguing and valuable for potential therapeutic applications of MLD in lumbar spine surgery; however, the sections in the introduction and results are not clearly presented, explained, or discussed.

Some improvements should be added to the manuscript to make it more accurate and coherent.

Major points:

- In order to enhance the manuscript's quality, I recommend adding a section in the introduction that explains why surgical treatment for adult lumbar spinal disorders carries a considerable risk of intraoperative and perioperative complications. The relationship between these complications and different risk factors, such as the patient's age, medical comorbidities, and the extent of the fusion up to the sacrum should also be elaborated upon.

- In section 3.2 of the paper, the authors discuss the analysis of participants' operational data. However, the presentation of the data in Figures 5 and 6 is imprecise, unclear, and inaccurate. Although each graph shows the value per Anteroposterior (AP), Lateral (Lat), Normal Position (N), and Rotated Position (CR), they are not discussed individually. To better understand the authors' conclusions on the shorter operation time (13.66±5.61 vs 25.63±8.17 min), fewer imaging attempts (15.05±7.84 vs 32.50±7.24), and smaller initial deviation (22.9±8.9% vs 61.5±15.7%), I suggest revising the paragraph and providing a more detailed description of the data presented in those figures.

- The manuscript lacks Tables 1, 2, and 3. Although the authors claim that all relevant data is included in the manuscript and its supporting information files, the tables are missing and only their captions are present. As a result, it was not possible to verify the data contained within the tables.

Minor points:

- I suggest revising the English form of the manuscript (e.g., line 68: change the "A questionnaire assessed the learning experience" to "A questionnaire was used to assess the learning experience")

- In the manuscript, some mistakes are present (e.g., line 93: change “C-arm fluoroscopy is critical **in** precise surgical localization” to “C-arm fluoroscopy is critical **for** precise surgical localization”. Line 108: Change “We hypothesized the MLD” to “We hypothesized t**hat** the MLD”).

- In the manuscript, some typos are present. I suggest a spell-check of the text. (e.g., line 254: “guidance[9, 10]”. Line 189: “imaging[11]”.

-Authors utilize many abbreviations in the manuscript, so I suggest adding a list of abbreviations that can facilitate the reading and understanding of the text.

-When an acronym is used for the first time in the manuscript, it should be spelled out in full (e.g., OEC One CFD).
